# Seroprevalence of Bluetongue Virus in Dairy Herds with Reproductive Problems in Sudan

**DOI:** 10.1155/2014/595724

**Published:** 2014-03-19

**Authors:** Amira Mohamed Elhassan, Mohamed Abdalla Fadol, Abdel Rahim Mohamed El Hussein

**Affiliations:** Veterinary Research Institute (VRI), P.O. Box 8067, El Amarat, Khartoum, Sudan

## Abstract

The objectives of this cross-sectional study were to determine the seroprevalence of blue tongue virus (BTV) and assess potential risk factors associated with BTV infection in dairy cattle with reproductive problems in Sudan. Serum samples were collected from a total of 784 animals from 37 herds and tested for antibodies against BTV using cELISA. A total of 663 out of 784 (84.57%) sera tested proved positive for BTV antibodies in all farms tested in Khartoum and Gazira States. The prevalence of antibodies was high in both areas being 94.32% in Gazira State and 76.62% in Khartoum State. BTV antibodies prevalence were significantly higher (*P* < 0.000) in older animals than in younger ones. These rates were also significantly higher in the rainy season (*P* < 0.000) and in Gazira State compared to Khartoum State. Sex also showed significant (*P* < 0.000) differences in the seroprevalence, whereby females (74.7%) had higher level than males (9.8%). However, no significant (*P* > 0.09) variations for BTV seroprevalence were observed between breeds. The BTV antibodies prevalence in infertility cases (86.6%) was not significantly different from that found in abortion (74.3%) or neonatal death (66.7%) cases. The high seroprevalence of BTV recorded herein calls for control strategy to be implemented.

## 1. Introduction

Because of the difficulties involved in the diagnosis of the causative agent, bovine fetal loss resulting from abortion and/or infertility can sometimes be challenging to farmers, veterinarian, and diagnostic laboratories [1, 2].

Several bacterial, viral, protozoan, and fungal pathogens had been associated with infertility and abortions in cattle [3]. Viral agents incriminated include bovine herpes-1 (BoHV-1), bovine viral diarrhea virus (BVDV), and bluetongue virus (BTV) [4–6].

Bluetongue virus is the prototype species of the genus* Orbivirus*, family Reoviridae and occurs almost globally between latitudes 35°S and 50°N. In infected animals, the disease is characterized by various clinical forms, with symptoms ranging from acute to subacute, mild, or inapparent. In addition, this virus is responsible for fetal death, congenital defects, and reproductive failure in cattle [6]. At least 24 immunologically distinct serotypes of the virus are recognized [7–9].

Cattle are readily susceptible to infection with BTV; bovine BTV infections are important because cattle can serve briefly as a source of infection for* Culicoides* species that biologically transmit BTV to sheep and other ruminants [10].

The sequelae of abortion and the birth of calves with congenital defects like dwarf-like conformation, crooked legs, and gingival hyperplasia may occur in cattle naturally infected with BTV in early stages of pregnancy [11].

The diversity of viral serotypes and the wide host range of BTV complicate the development of diagnostic procedures. General types of tests that have primarily been used in diagnosis of BTV infection such as the complement fixation test, the agar gel immunodiffusion (AGID) test, and the more current ELISA tests are useful in detecting animals that have had prior exposure to BTV [12, 13].

In Sudan, BTV was first detected in 1953 when an outbreak occurred in sheep in the Gazira research farm in Wad Medani, Central Sudan. The presence of BTV in these samples was confirmed by the Veterinary Research Laboratories at Onderstepoort [14]. Subsequently, serological surveys showed that antibodies against BTV were widespread in livestock species such as domestic species including sheep, goats, cattle, and camels in the country [15]. Several virus serotypes were isolated from outbreaks of sheep, from apparently healthy cattle, and from* Culicoides* midges [16–18].

The aim of this investigation was to determine the prevalence of BTV antibodies among Sudanese dairy herds with reproductive disorders and assess the risk factors involved.

## 2. Materials and Methods

### 2.1. Study Area

This survey was conducted in Khartoum and Gazira States. The investigation area extends from the latitudes 43° 20′E to 32° 50′W and longitudes 15° 75′N to 13° 32′S. This area is semiarid in the north and savannah in the south. Serum samples were collected from 37 herds with a history of reproductive problems (17 herds in Khartoum State and 20 herds in Gazira State) (Table 1). Animals sampled included individuals with history of abortion, infertility, and neonatal calf death or animals with no such history. Animals of both sexes and various age groups (less than 1 year, 1-2 years, and above 2 years old) were sampled in different seasons: winter (November–February), summer (March–June), and rainy season (July–October).

### 2.2. Serum Samples

Blood samples were collected from 784 local and cross bred animals. Samples were centrifuged and the serum was kept at −20°C until assayed for the presence of antibodies against BTV.

### 2.3. Individual Animals with Recognized Reproductive Problems

Of the 784 animals sampled, 120 were identified according to their owners, having reproductive problems such as infertility, abortion, and neonatal death. The data from these animals were analyzed to assess association of these problems with BTV antibody prevalence.

### 2.4. ELISA Test

A commercial competitive enzyme linked immunosorbent assay (cELISA) kit (Investcare Vet Company, VMRD, USA) was used to detect antibodies against BTV according to manufacturer's instructions. A herd was considered positive when at least one animal showed positive reaction.

### 2.5. Statistical Analysis

The serological results and other information such as locality, age, sex, season, and breed (indigenous and cross bred) of the sampled animals were edited and analyzed using statistical package (SPSS version 16). To identify the associations of the risk factors with BTV seroprevalence, the Chi-square (*χ* ^2^ test) was used. The statistical significance level used was *P* ≤ 0.05.

## 3. Results

### 3.1. BTV Seroprevalence at Herd and General Individual Level

Antibodies against BTV could be detected in 663 (84.6%) of 784 sera samples tested with the cELISA. BTV antibodies were detected in all farms tested in Khartoum and Gazira States (Table 1). However, the prevalence of antibodies was significantly higher (94.32%, *P* < .000) in Gazira State than Khartoum State (76.62%) (Table 2).

The seroprevalence of BTV antibodies was also significantly different among age groups with 1-2-year-old and above-2-year-old animals revealing significantly (*P* < 0.000) higher seroprevalence than younger ones. The seroprevalence was also significantly higher during the rainy season (*P* < 0.000) and in females (*P* < 0.000) compared to males (Table 3). However, no significant (*P* > 0.09) variations for BTV antibodies seroprevalence were observed between breeds of animals (Table 3).

### 3.2. BTV Seroprevalence in Animals with Recognized Reproductive Problems

The highest prevalence (86.6%) of BTV antibodies detected in cases of infertility was not significantly (*P* ≤ 0.2) higher than that of other reproductive problems (Table 4). However, the overall seroprevalence (77.5%) observed in these animals was lower than the overall prevalence (84.6%) observed at the general herd level survey (Tables 1 and 4).

## 4. Discussion

Results of the present investigation corroborated earlier serological and virological evidence for widespread BTV infection in dairy cattle in Sudan. BTV antibodies were prevalent both at herd and individual animal level and were detected in all farms tested in Gazira and Khartoum States. However, prevalence rates in the present study were generally higher than those observed in previous studies [15, 19, 20]. This discrepancy may partly be ascribed to the apparent greater sensitivity of the cELISA used in the present study compared to that of the AGID technique used in earlier studies.

During this investigation, there were significant differences between prevalence of antibodies to BTV among various age groups. Animals older than 1 year had significantly higher prevalence rates (*P* ≤ 0.000) than younger ones. The prevalence of BTV antibodies was also significantly higher during rainy season (*P* ≤ 0.000) compared to other seasons and in Gazira State (*P* ≤ 0.000) compared to Khartoum State (Tables 1 and 2). This may be due to the higher abundance of the vector (*Culicoides species*) during the rainy season. The irrigation scheme at Gazira, consisting of a vast number of irrigation canals, may enhance the abundance and survival of the vector which will lead to higher rates of virus transmission. Variations in seasonal incidence of BTV in Central Sudan were mentioned by Mohamed and Mellor to be due to the seasonal prevalence of midges (*Culicoides imicola*), whose population increased steadily through the rainy season. No significant variations for BTV antibodies prevalence were observed (*P* ≤ 0.09) between indigenous and cross breed animals [21].

The results observed herein for BTV antibody prevalence in relation to season, age groups, and breed are in agreement with those of Uhaa et al. [22] in California (USA). On the other hand, in our study, sex was a highly significant (*P* ≤ 0.000) risk factor and females were more prone to infection than males. Although it is hard to explain, this might be due to a sample size bias originating from the availability of animals on the farms.

Examination of the 120 serum samples collected from animals that apparently suffered from reproductive problem including abortion, infertility, and death after birth cases revealed that BTV antibodies were highly (86.6%) prevalent in cases of infertility with no significant variations (*P* ≤ 0.2). Vandaele et al. [23] stated that embryonic apoptosis after BTV-8 infection results in embryonic arrest and early embryonic death and thus might be involved in herd fertility problems during a BTV epidemic. This may be explained by the time when pregnant cattle get infected. If the infection occurred during the first 100 days of gestation, the conception may be resorbed, aborted, or may result in stillbirths, birth of week calves, or birth of calves with cerebral abnormalities and this is usually associated with serotypes 10, 11, 13, and 17. After 150 days of gestation, infection had negative effects on fetuses [3, 24].

However, the results also revealed that the overall seroprevalence (77.5%) observed in animals which suffer reproductive problem was lower than the overall prevalence (84.6%) observed in the general survey. This indicates that other agents may be involved in these problems. Indeed, earlier studies have shown that agents such as bovine viral diarrhea virus, bovine herpes viruses 1 and 4, Akabane virus and* Neospora caninum* were highly prevalent in cattle in Sudan [25–27].

## 5. Conclusion 

The seroprevalence for the BTV antibodies was high in the two geographic areas studied. This may also be at risk to other pathogens transmitted by* Culicoides* in Sudan, since the apparent aridity of the area does not hamper the transmission of* Culicoides* borne diseases. The seroprevalence BTV was significantly associated with older age, location, season, and sex; however, no clear association was discerned regarding abortion, infertility, or neonatal death. The high prevalence of BTV antibodies reported here indicates that BTV is highly endemic in Sudan. Further investigations on the virology and epidemiology should be initiated to gather information necessary for establishing national control strategy for BTV in Sudan. In general, this strategy should include use of appropriate vaccine(s), vector control, and sound management practices. In addition, movements of livestock germplasm into Sudan should consider freedom from this disease agent as outlined by international codex of animal trade in order to prevent introduction of new virus serotypes.

## Figures and Tables

**Table 1:** Herds levels of infection with bluetongue virus in cattle in Sudan.

States	Location	Number of herds	Number of positive herds (%)
Khartoum	Khartoum	5	5 (100)
	Khartoum North	7	7 (100)
	Omdurman	5	5 (100)

Gazira	Alkamleen	1	1 (100)
	Wad Medani	17	17 (100)
	Sennar	1	1 (100)
	Kenana	1	1 (100)

Total		37	37 (100)

**Table 2:** Seropositivity to BTV in cattle from different locations in Khartoum and Gazira States.

State	Location	Sample	Number of positive samples (%)	*P* value
Khartoum	Khartoum	123	108 (87.8%)	
	Khartoum North	210	180 (85.71%)	
	Omdurman	99	43 (43.43%)	
	Subtotal	432	331 (76.62%)	

Gazira	Alkamleen	12	11 (91.67%)	
	Wad Medani	327	308 (94.19%)	
	Sennar	7	7 (100%)	
	Kenana	6	6 (100%)	
	Subtotal	352	332 (94.32%)	0.000

	Total	784	663 (84.6%)	

**Table 3:** Influence of some risk factors on seroprevalence of bluetongue virus (BTV) in Sudan.

Factor	Group	Number of tested animals	Number of positive animals (%)	*P* value
Breed	Local	47	36 (76.5)	>0.05
	Cross	737	627 (85.07)	

Sex	Female	673	586 (87.07)	0.000
	Male	111	77 (69.3)	

Season	Winter	141	98 (69.5)	0.000
	Dry	169	124 (73.3)	
	Rainy	474	441 (93.3)	

Age	Less than 1 year	143	94 (65)	0.000
	1-2 years	158	141 (89.2)	
	Above 2 years	483	428 (88.6)	

**Table 4:** Prevalence of BTV antibodies in animals afflicted with abortion, infertility, and death of calves after birth in Sudan.

Status	Number of sera tested	Numbers of positive sera	*P* value
Abortion	70	52 (74.3%)	0.2
Infertility	38	33 (86.8%)	
Death after birth	12	8 (66.7%)	

Total	120	77.5%
